# Pulmonologist-Performed Ultrasound-Guided Fine-Needle Aspiration of Lung Lesions

**DOI:** 10.3390/reports7020026

**Published:** 2024-04-10

**Authors:** Chin-Tong Kwok, Yiu-Cheong Yeung, Yu-Hong Chan, Man-Ying Ho

**Affiliations:** Department of Medicine and Geriatrics, Princess Margaret Hospital, Hong Kong, China; yeungyc@ha.org.hk (Y.-C.Y.); cyh423a@ha.org.hk (Y.-H.C.); hmy069@ha.org.hk (M.-Y.H.)

**Keywords:** pulmonologists performed, ultrasound guided, fine-needle aspiration, peripheral lung lesion

## Abstract

Background and objective: Lung cancer is increasingly common, and accurate diagnosis is important for personalized treatment. Ultrasound-guided percutaneous fine-needle aspiration (FNA) is a useful method to obtain a specimen for a histological diagnosis of peripheral lung lesions. The aim of this study is to evaluate the diagnostic accuracy and complication rate of the procedure performed by pulmonologists. The result is compared with that of ultrasound-guided core needle biopsy performed by radiologists. Methods: We retrospectively evaluated the diagnostic accuracy and complication rates of pulmonologist-performed ultrasound-guided FNAs of lung lesions in the period of 1 August 2019 to 30 June 2021 (pulmonologist group) and radiologist-performed ultrasound-guided core needle biopsies (CNBs) of lung lesions in the period of 1 January 2010 to 31 December 2014 (radiologist group). A logistic regression analysis was used to identify independent influence factors associated with diagnostic accuracy in the pulmonologist group and in the combination of both groups. Results: In a 23-month period, pulmonologists in a tertiary center performed 113 episodes of ultrasound-guided fine-needle aspiration for peripheral lung lesions. The diagnostic accuracy and complication rates were 80.4% and 5.3%, respectively, compared to 86.8% and 7.4% in a historical cohort consisting of 68 episodes of ultrasound-guided core needle biopsies performed by radiologists in the same hospital. Lung lesions located in the upper lobe were predictive of successful diagnoses. Conclusions: An ultrasound-guided fine-needle aspiration by a pulmonologist is an easily accessible and reliable method to obtain specimens for histological diagnoses.

## 1. Introduction

Lung mass is a common finding in thoracic imaging [1]. It is expected that pulmonologists will receive an increasing number of referrals for lung masses, which poses a challenge regarding resources for diagnostic procedures. The American College of Chest Physicians’ evidence-based clinical practice guidelines on the diagnosis and management of lung cancer recommend that the diagnosis should be confirmed using the least invasive method [2]. Typically, a histological diagnosis is obtained by bronchoscopic methods for centrally located lung lesions [3] and by image-guided percutaneous biopsies for peripherally located lung lesions [4]. For a lung lesion abutting the chest wall without an aerated lung between the lesion and the pleura, ultrasound serves as a good imaging method [5]. An ultrasound-guided fine-needle aspiration (FNA) of a lung lesion was proven to be safe in the hands of pulmonologists [6]. It is a reliable method to obtain histology [7], even in patients with cancer complications such as superior vena cava syndrome [8]. We retrospectively evaluated the diagnostic accuracy and complication rates of pulmonologist-performed ultrasound-guided FNAs of lung lesions in the period of 1 August 2019 to 30 June 2021 (pulmonologist group) and radiologist-performed ultrasound-guided core needle biopsies (CNBs) of lung lesions in the period of 1 January 2010 to 31 December 2014 (radiologist group).

Our objective was to evaluate the diagnostic accuracy and complication rates of pulmonologist-performed ultrasound-guided fine-needle aspirations and radiologist-performed ultrasound-guided core needle biopsies of peripheral lung lesions.

Our secondary objective was to analyze the factors that predict diagnostic accuracy by ultrasound-guided FNAs and CNBs of lung lesions.

## 2. Methods

### 2.1. Subjects

The full list of patients in the pulmonologist group was kept by the pulmonologists. The historical control of radiologist-performed ultrasound-guided CNBs of lung lesions in the period of 1 January 2010 to 31 December 2014 (radiologist group) was chosen for comparison of diagnostic accuracy and complication rate. The list of patients in the control group was obtained by searching the Clinical Data Analysis and Reporting System (CDARS) in Hospital Authority with the keywords ‘fine needle aspiration biopsy of lung’ and ‘percutaneous fine needle aspiration biopsy of lung with imaging guidance’ (ICD code 33.26). Among the searched patients, cases performed by radiologists under ultrasound guidance would be recruited in the radiologist group as the historical control.

### 2.2. Diagnostic Category

Cases of ultrasound-guided sampling of lung lesions were classified as true positive, true negative, false positive and false negative. Cases with histological diagnoses of specific neoplastic conditions, infectious diseases or benign conditions which had consistent clinical courses for 6 months after the procedures were defined as true positive. False negative was defined as necrotic material, quantity insufficiency, atypical cells or suspicious of malignancy in the FNA result, but the definitive diagnosis was subsequently made using other methods such as bronchoscopy, computed tomography (CT)-guided biopsy, surgical biopsy or microbiological culture. True negative was defined as non-diagnostic cytology with stable or receding lesions at follow-up. Cases with clinical courses that were inconsistent with tissue sampling suggested that the diagnoses were regarded as false positive. The diagnoses were categorized as primary lung cancers (with different histological subtypes), metastases, hematological malignancies, malignancies not otherwise specified, infections, benign lesions and non-diagnostic.

### 2.3. Study Design

Diagnostic accuracy is defined as the proportion of true positives and true negatives in all evaluated cases. The sensitivity, specificity, positive predictive value and negative predictive value would be calculated. Complications consist of pneumothorax and hemoptysis. Pneumothorax is defined as the presence of air in the ipsilateral pleural cavity on a chest X-ray within 24 h after the procedure. Hemoptysis is defined as patient-reported blood-stained sputum within 24 h after FNA. The predictive factors that would be analyzed include age, gender, smoking status, lesion size and location.

#### Statistical Analysis

The 95% confidence interval and *p*-value of diagnostic accuracy, sensitivity, specificity, positive predictive value and negative predictive value of both the pulmonologist and radiologist groups were calculated. Pearson’s chi-squared test and Fisher’s exact test were employed to test for differences between groups of categorical variables. Independent *t*-test and Mann–Whitney U test were used to test for differences between groups of continuous variables. Logistic regression analysis was used to identify independent influence factors associated with diagnostic accuracy in pulmonologist group and in combination of both groups. Statistical analysis was conducted using SPSS version 27. A *p*-value < 0.05 was considered statistically significant.

### 2.4. The Ultrasound-Guided Fine-Needle Aspiration

**Figure 1:**
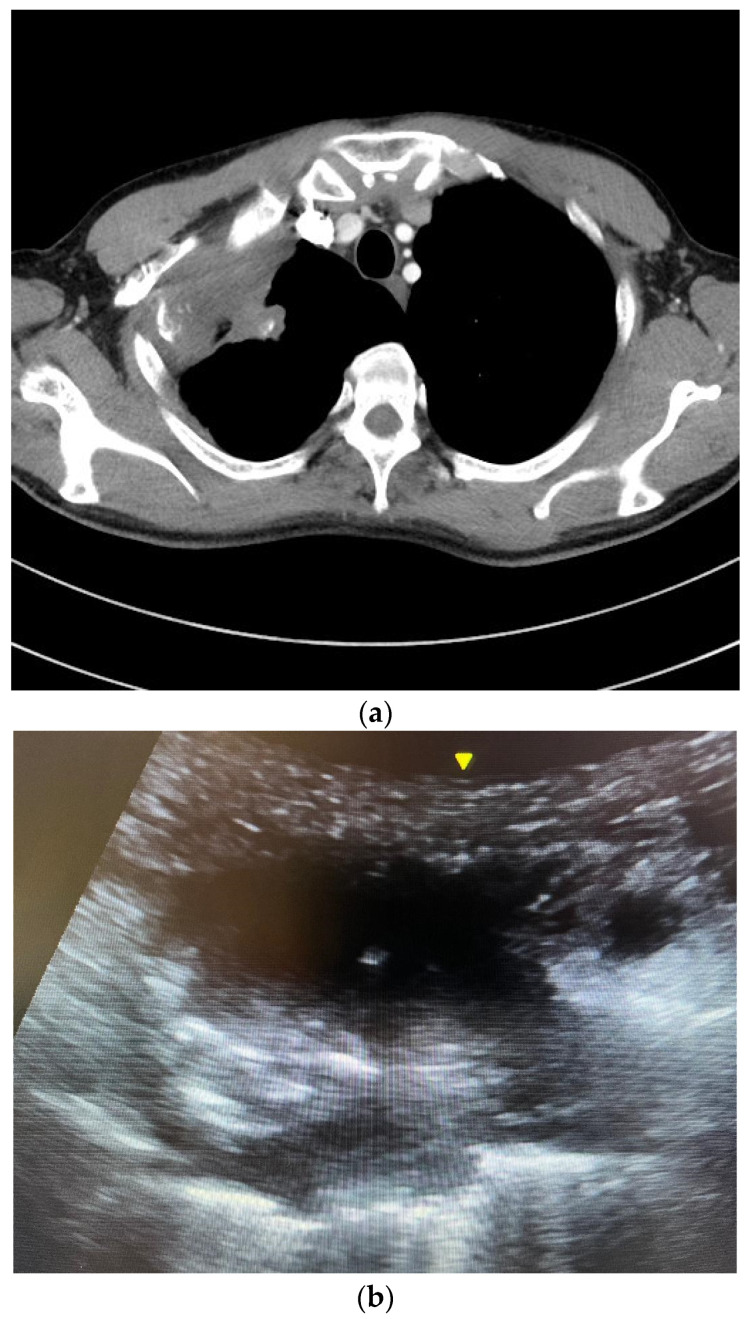

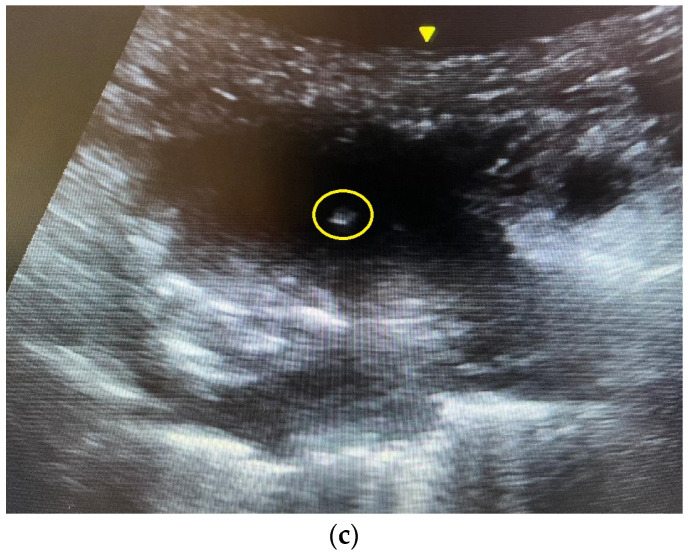
(**a**) A computed tomography image showing a right upper lobe Pancoast tumor. The tumor closely abutted the chest wall, allowing ultrasound-guided percutaneous biopsy. The pathology was squamous cell carcinoma. (**b**) An ultrasound image of the Pancoast tumor. The tumor had soft tissue density with heterogeneous echogenicity. (**c**) The yellow circle indicated the position of the needle tip inside the tumor. Several in and out motions were made with the needle, and the needle tip’s position was traced inside the tumor under real-time ultrasound guidance.

Patients were instructed to sit upright, lie supine or lie lateral. The ultrasound machine Venue 40^®^ or Venue 50^®^ (General Electric, Boston, MA, USA) with curvilinear probe was used to locate the lesion. Color Doppler was employed to check for vessels running through the lesion. The operators would avoid puncturing the vessels during FNA. Under real-time ultrasound guidance and aseptic technique, 5 mL 2% lignocaine was injected percutaneously for local anesthesia. A 22-gauge (22 G) Spinocan^®^ spinal needle (B. Braun, Melsungen, Germany) was inserted, and the needle tip’s position within the target lung lesion was confirmed using a real-time ultrasound exam in the short axis or long axis (Figure 1). The needle stylet was then removed, and a 20 mL syringe was attached to the needle. Four to five in and out motions were made with the needle. At the same time, suction was applied via the 20 mL syringe. Cytological material obtained was injected into a formalin bottle. At least two punctures were made.

Cell blocks were prepared in the pathology laboratory using agar cell blocks to retain tissue fragments. The agar cell blocks were then subjected to tissue processing sequence. The tissue specimens were reviewed by pathologists. Rapid on-site examination was not available in our center. After the FNA or biopsy, an ultrasound was performed again to look for any disappearance of the lesion under pleura, suggesting the presence of immediate pneumothorax. Standard chest X-ray was performed 4 h after the procedure to look for pneumothorax. The patient was monitored hourly for 4 h in the hospital and was discharged if no complication arose.

For radiologist-performed ultrasound-guided core needle biopsy of lung masses, core biopsy was performed similarly as fine-needle aspiration but with needles of different sizes and from different companies (Table 1 and Table 2).

## 3. Results

There were 105 patients with 113 episodes of pulmonologist-performed ultrasound-guided FNAs of lung lesions that were on the list kept by pulmonologists. The CDARS search for radiologist-performed image-guided CNBs yielded 391 results. Computed tomography was used as the image guidance method in 323 cases. A total of 65 patients with 68 ultrasound-guided CNBs performed by radiologists were included in the radiologist group as the historical cohort.

### 3.1. Baseline Characteristics and Lung Lesion Profile

There was no statistically significant differences in gender, smoking status and lung lesion size between the two groups. There were statistically significant differences between the two groups in age (*p*-value = 0.02) and lung lesion location (*p*-value = 0.049). The mean ages were 68.7 and 64.4 in the pulmonologist group and radiologist group, respectively. The lung lesion locations are charted in Table 3.

### 3.2. Diagnostic Accuracy and Yield

In the pulmonologist group, one episode of FNA was excluded for the diagnostic accuracy analysis because the patient died from pneumonia one month after the procedure. In the remaining 112 episodes of FNAs, 88 episodes (79%) were regarded as true positive, and 2 episodes (2%) were regarded as true negative. There were 22 false negative episodes (19%) and no false positive episodes. The diagnostic accuracy was 80.4% (95% CI 72–86.7%). The sensitivity was 80% (95% CI 71.6%–86.4%), and the negative predictive value was 8.3% (95% CI 2.3%–25.8%). The specificity and positive predictive value were both 100% (Table 4).

Among the 68 episodes of CNBs in the radiologist group, 57 episodes (84%) were regarded as true positive, and 2 episodes (3%) were regarded as true negative. There were 9 false negative episodes (13%) and no false positive episodes. The diagnostic accuracy was 86.8% (95% CI 76.7–92.9%). The sensitivity was 86.4% (95% CI 76.1%−92.7%), and the negative predictive value was 18.2% (95% CI 5.1%−47.7%). The specificity and positive predictive value were both 100% (Table 4).

The diagnostic accuracy (80.4% vs. 86.8%, *p*-value = 0.27), sensitivity (80% vs. 86.4%, *p*-value = 0.283) and negative predictive value (8.3% vs. 18.2%, *p*-value = 0.575) of the pulmonologist and radiologist groups were comparable. The results are summarized in Table 4.

### 3.3. Complication Rates

The overall complication rates for the pulmonologist group and radiologist group were 5.3% and 7.4%, respectively (*p*-value = 0.749). One patient in each group who developed pneumothorax after the procedure required chest drain insertion, while the other patients were managed conservatively with oxygen supplementation (Table 5).

### 3.4. The Diagnoses

The diagnoses by FNAs or CNBs were categorized as primary lung cancers (119, 65.7%), metastases (6, 3.3%), hematological malignancies (7, 3.9%), malignancies not otherwise specified (3, 1.7%), infections (8, 4.4%), benign lesions (3, 1.7%) and non-diagnostic (35, 19.3%) (Table 6).

### 3.5. Predictive Factors

In the pulmonologist group, univariable regression (with ridge penalty) showed that the lesion location of upper lobe was predictive of the diagnostic accuracy of ultrasound-guided FNA (OR = 5.32, 95% CI 1.78–15.87; *p*-value = 0.002) (Table 7). When combining the pulmonologist and radiologist groups, the univariable regression showed that both the lesion size (OR = 1.17, 95% CI 1.0–1.38; *p*-value = 0.048) and lesions located in the upper lobe (OR = 4.96, 95% CI 1.97–12.48; *p*-value < 0.001) were predictive of the diagnostic accuracy of ultrasound-guided percutaneous tissue sampling (Table 8). Multivariable regression showed that only lesions located in the upper lobe (OR = 4.42, 95% CI 1.73–11.28; *p*-value = 0.002) were predictive of the diagnostic accuracy of ultrasound-guided percutaneous tissue sampling (Table 8).

### 3.6. Genetic Mutation Analysis

A genetic mutation analysis, including EGFR, Anaplastic Lymphoma Kinase (ALK) and Reactive Oxygen Species (ROS) mutation, with or without the Programmed Cell Death Ligand 1 (PDL1) test, were requested in 61 cases in the pulmonologist group and in 21 cases in the radiologist group, respectively. FNA tissue was adequate for analysis in 59 cases (96.7%) and inadequate in 2 cases (3.3%) in the pulmonologist group. Biopsy specimen was adequate for analysis in 19 cases (90.5%) and inadequate in 2 cases (9.5%) in the radiologist group.

## 4. Discussions

Our study showed that pulmonologist-performed ultrasound-guided FNAs of lung lesions had a diagnostic accuracy of 80.4% and a complication rate of 5.3%, which were comparable to a historical cohort of radiologist-performed ultrasound-guided core needle biopsies of lung lesions in the same hospital (86.8% and 7.4%, respectively) (Table 4).

### 4.1. Diagnostic Accuracy in the Literature

Pulmonologists in different countries performed ultrasound-guided percutaneous tissue sampling with diagnostic yield ranging from 72% [9] to 95% [10] for fine-needle aspiration [9,10,11,12] and from 83% [13] to 93.4% [14] for core needle biopsy [6,12,13,14]. The diagnostic yield is 70.9% [7] for case series consisting mostly of lung lesions located in the parenchyma. Our study showed a diagnostic accuracy of 80.4%, which was comparable to that in the literature. The diagnostic yield was similar to that for biopsies performed by pulmonologists and radiologists in the literature [12]. The site of the biopsy is not limited to the peripheral lung and pleura, but also includes other intrathoracic areas such as the rib, mediastinum and chest wall in the literature [7,12,15]. From our experience, FNAs of the rib, cervical lymph node and mediastinal lesions were also feasible but not included in this study.

### 4.2. The Complication Rate in the Literature

Complications of ultrasound-guided FNAs of peripheral lung lesions include pneumothorax and hemoptysis. The overall complication rate was quoted between 2.7% and 15% in the literature [7,11,12,13,14]. The most common complication is pneumothorax, rated from 0% to 7.9% [6,7,13,14]. Chest drain insertion and hospital admission are rarely needed for these complications [11]. The complication rate of 5.3% in the pulmonologist group in our study highlights the procedure’s safety.

### 4.3. Predictive Factors of Diagnostic Yield

A large study involving more than 9000 patients in Korea regarding percutaneous image-guided biopsies of lung lesions showed that the risk factors for diagnostic failure are a lesion size smaller than 2 cm, subsolid lesions, FNAs without CNBs and a final diagnosis of benign lesions and lymphoma [16]. For ultrasound-guided FNA, factors associated with diagnostic success include the suspicion of malignancy, an increased size and a lack of pleural sliding, which are suggestive of pleural adhesion [11].

In our series, a larger lesion size and upper lobe location increased the diagnostic yield (Table 7 and Table 8). The lesion size is a well-established factor for diagnostic yield. A lesion size of at least 2 cm is appropriate for ultrasound-guided FNA. Our study is the first to identify that upper lobe location is predictive of the diagnostic accuracy. There are two possible reasons for a higher diagnostic rate in the upper lobe than in other lobes. Firstly, the magnitude of lung tumor movement during respiration is larger in the lower lobe than in the upper lobe [17,18]. As tissue sampling takes around 10 s and patients often cannot hold their breaths throughout, lesion movement with respiration may lead to a missed target. Secondly, lung lesions in the upper lobe are more likely to be malignant than those in other lobes [19]. Upper lobe location is a cancer predictor in lung nodules detected in screening CTs [20], and this was validated in prediction models such as the Brock University cancer prediction equation. The diagnostic accuracy of a malignant lesion by FNA is much higher than a benign lesion [21]. Therefore, a lesion located in the upper lobe is a positive predictive factor of the diagnostic accuracy by FNA.

### 4.4. Strengths

Our study had several strengths. Firstly, this is the first local study demonstrating that pulmonologist-performed ultrasound-guided FNAs of lung lesions have comparable diagnostic accuracy to that in the literature. Secondly, we demonstrated a comparable diagnostic accuracy between FNA and CNB. Thirdly, a low complication rate of the procedure was recorded, ensuring patient safety. 

### 4.5. Limitations

This study has several limitations. Firstly, this study is a single-center retrospective study comparing the pulmonologist group with the radiologist group, which was a historical cohort. Chronological bias existed. Advancement in imaging technology, equipment and pathological diagnostic technique may favor the pulmonologist group. Secondly, there was selection bias in both the pulmonologist group and radiologist group. Difficult cases such as small lesions might be referred by pulmonologists to radiologists for CT-guided biopsy. The radiologists had variable preferences on the choice between CT and ultrasound guidance. Thirdly, there were statistically significant differences in the demographics between the pulmonologist group and the radiologist group in age and lung lesion location. The pulmonologist group cohort was older than that of the radiologist group. We postulated that ultrasound-guided FNA was a readily accessible bedside procedure and was thus more acceptable to the elderly when compared with a scheduled CNB by radiologists. The *p*-value for lesion location difference is 0.049, which is only marginally significant. Fourthly, there were variations in the equipment, technique, operators’ experience and peri-procedure practices. The procedures were performed by different operators with various experiences in this study.

### 4.6. Further Development

In our cohort, a patient with an 8 cm right middle lobe lung mass consisting of central necrosis received five sessions of percutaneous ultrasound-guided FNAs and biopsies, three of which were performed by pulmonologists and two by radiologists from two different hospitals. All five specimens showed necrotic materials suspicious of malignancy only. The patient subsequently refused further invasive procedures and died without a definitive diagnosis. This case highlights the usefulness of contrast-enhanced ultrasound in distinguishing necrosis, atelectasis and tumors. Lungs receive blood supply mainly from pulmonary arteries. Lung tumors receive blood supply mainly from the bronchial artery, while the necrotic area receives minimal blood supply. Contrast enhancement for an atelectatic lung is short and marked, while that of a tumor is delayed and variable [22]. Differentiating areas composed of a tumor and of necrosis or atelectasis is therefore possible by analyzing the arrival time of the contrast and the extent of enhancement. A study employing contrast-enhanced ultrasound in tissue sampling of a lung mass with atelectasis showed promising results [23]. The contrast agent (SonoVue^®^) is available in centers that offer contrast echocardiography services. In selected cases with large areas of necrosis or an atelectatic lung, contrast-enhanced ultrasound can guide accurate tissue sampling.

## 5. Conclusions

Ultrasound-guided FNAs of peripheral lung lesions by pulmonologists is an accurate and safe procedure. Lung lesions located in the upper lobe are predictive of diagnostic success. Pulmonologists should be encouraged to perform this procedure to achieve early diagnoses.

## Figures and Tables

**Table 1:** Biopsy needle type used by radiologists.

Needle Type	Count
Temno	40.5
Vac-Cut	20.5
Magnum	3
Chiba	2
Ultracore	1
Spinal	0.5
Coaxial	0.5

Radiologists used two types of needles in one session in some occasions, and the number was counted as 0.5 for each needle.

**Table 2:** Biopsy needle size used by radiologists.

Needle Size	Count
17 G	1
18 G	45.5
19.5 G	6
20 G	10
21 G	2.5
22 G	3

Radiologists used two types of needles in one session in some occasions, and the number was counted as 0.5 for each needle.

**Table 3:** Baseline characteristics and lung lesion profiles of patients.

	Pulmonologist Group (*n* = 113)	Radiologist Group (*n* = 68)	*p*-Value
Age, year, mean ± SD [range]	68.7 ± 12 [26–94]	64.4 ± 12.6 [31–84]	**0.020**
Gender (male/female)	85:28	55:13	0.378
Smoking status, n (%)			0.558
Non-smoker	35 (31)	22 (32.5)	
Ex-smoker	33 (29)	24 (35)	
Smoker	45 (40)	22 (32.5)	
Lung lesion size, centimeter, mean ± SD [range]	6.5 ± 2.9 [1.7–19]	6.7 ± 3.2 [2–17]	0.687
Lung lesion location, n (%)			**0.049**
Right upper lobe	40 (35)	20 (29.4)	
Right middle lobe	7 (7)	3 (4.4)	
Right lower lobe	26 (23)	16 (23.5)	
Left upper lobe	17 (15)	17 (25)	
Left lingula lobe	0	4 (5.9)	
Left lower lobe	23 (20)	8 (11.8)	

**Table 4:** Diagnostic accuracy and yield.

	Pulmonologist Group (*n* = 112)	Radiologist Group (*n* = 68)	*p*-Value
Accuracy (95% CI)	80.4% (72.0%−86.7%)	86.8% (76.7%−92.9%)	0.270
Sensitivity (95% CI)	80% (71.6%−86.4%)	86.4% (76.1%−92.7%)	0.283
Specificity (95% CI)	100% (34.2%−100%)	100% (34.2%−100%)	1
Positive predictive value (95% CI)	100% (95.8%−100%)	100% (93.7%−100%)	1
Negative predictive value (95% CI)	8.3% (2.3%−25.8%)	18.2% (5.1%−47.7%)	0.575

**Table 5:** Complication rate.

	Pulmonologist Group (*n* = 113)	Radiologist Group (*n* = 68)	*p*-Value
Any complication, n (%)	6 (5.3%)	5 (7.4%)	0.749
Pneumothorax, n (%)	6 (5.3%)	4 (5.9%)	1
Hemoptysis, n (%)	0	1 (1.5%)	0.376

**Table 6:** Diagnoses by fine-needle aspiration or core needle biopsy (total *n* = 181).

	Pulmonologist Group (*n* = 113)	Radiologist Group (*n* = 68)
Lung cancer		
Adenocarcinoma	44 (39%)	18 (26%)
Squamous cell carcinoma	14 (12%)	14 (21%)
Non-small-cell carcinoma	10 (9%)	3 (4%)
Small cell carcinoma	6 (5%)	3 (4%)
SMARCA4-deficient malignant tumor	1 (1%)	0
Sarcomatoid carcinoma	0	2 (3%)
Lymphoepithelioma-like carcinoma	2 (2%)	1 (1%)
Adenosquamous carcinoma	1 (1%)	0
Metastasis	1 (1%)	5 (7%)
Hematological malignancies		
Lymphoma	3 (3%)	2 (3%)
Leukemic infiltrate	1 (1%)	1 (1%)
Malignancy not otherwise specified		
Malignant cells	1 (1%)	0
Carcinoma	1 (1%)	0
Spindle cell lesion	0	1 (1%)
Infection		
Aspergillosis	1 (1%)	2 (3%)
Cryptococcal infection	0	1 (1%)
Tuberculosis	3 (3%)	1 (1%)
Benign lesions		
Fibrous tumor	0	2 (3%)
Neurilemmoma	0	1 (1%)
Non-diagnostic		
Acute inflammation	2 (2%)	0
Necrosis	3 (3%)	1 (1%)
Suspicious cells	3 (3%)	1 (1%)
Atypical cells	2 (2%)	1 (1%)
Quantity insufficiency	7 (6%)	1 (1%)
Negative	7 (6%)	7 (10%)

**Table 7:** Predictive factor of diagnostic accuracy in pulmonologist group.

	Univariable Regression		
	OR_unadj_ (95% CI)		*p*
Lesion size	1.12	(0.93–1.34)	0.230
Lesion location ^a^			
Lower (ref.)	1		—
Middle/lingular	44.67	(0.18–11,240.83)	0.173
Upper	5.32	(1.78–15.87)	0.002
Age	1.01	(0.98–1.05)	0.475
Male	1.53	(0.55–4.26)	0.412
Smoking status			
Never (ref.)	1		—
Ex	1.28	(0.39–4.21)	0.680
Current	1.37	(0.46–4.11)	0.574

^a^ with ridge penalty.

**Table 8:** Predictive factors of diagnostic accuracy.

	Univariable Regression			Multivariable Regression		
	OR_unadj_ (95% CI)		*p*	OR_adj_ (95% CI)		*p*
Lesion size, cm	1.17	(1.00–1.38)	0.048	1.12	(0.95–1.31)	0.176
Lesion location						
Lower (ref.)	1		—	1		—
Middle/lingular	1.48	(0.38–5.85)	0.575	1.39	(0.35–5.56)	0.638
Upper	4.96	(1.97–12.48)	<0.001	4.42	(1.73–11.28)	0.002
Age, year	1.01	(0.98–1.04)	0.475	—		—
Male	1.81	(0.77–4.24)	0.171	—		—
Smoking status						
Never (ref.)	1		—			
Ex	1.09	(0.44–2.73)	0.852	—		—
Current	1.97	(0.74–5.21)	0.174	—		—

## Data Availability

The original contributions presented in the study are included in the article, further inquiries can be directed to the corresponding author.

## Abbreviations

ALK	Anaplastic Lymphoma Kinase
CDARS	Clinical Data Analysis and Reporting System
CI	Confidence Interval
CNB	Core Needle Biopsy
CT	Computed Tomography
EGFR	Epidermal Growth Factor Receptor
FNA	Fine-Needle Aspiration
ICD	International Code of Disease
OR	Odds Ratio
PDL1	Programmed Cell Death Ligand 1
